# Early Safety Findings Among Persons Aged ≥60 Years Who Received a Respiratory Syncytial Virus Vaccine — United States, May 3, 2023–April 14, 2024

**DOI:** 10.15585/mmwr.mm7321a3

**Published:** 2024-05-30

**Authors:** Anne M. Hause, Pedro L. Moro, James Baggs, Bicheng Zhang, Paige Marquez, Michael Melgar, Amadea Britton, Erin Stroud, Tanya R. Myers, Jeffrey Rakickas, Phillip G. Blanc, Kerry Welsh, Karen R. Broder, John R. Su, David K. Shay

**Affiliations:** ^1^Division of Healthcare Quality Promotion, National Center for Emerging and Zoonotic Infectious Diseases, CDC; ^2^Coronavirus and Other Respiratory Diseases Division, National Center for Immunization and Respiratory Diseases, CDC; ^3^Food and Drug Administration, Silver Spring, Maryland.

**Summary**

**What is already known about this topic?**

The Food and Drug Administration licensed Arexvy and Abrysvo vaccines in May 2023 for prevention of respiratory syncytial virus (RSV) lower respiratory tract disease in adults aged ≥60 years. In trials, Guillain-Barré syndrome (GBS) was identified as a potential safety concern.

**What is added by this report?**

Findings are consistent with those from trials; reports of GBS (4.4 and 1.8 reports per million doses of Abrysvo and Arexvy vaccine administered, respectively) were more common than expected background rates.

**What are the implications for public health practice?**

The Advisory Committee on Immunization Practices (ACIP) recommends adults aged ≥60 years may receive 1 dose of RSV vaccine. Population-based surveillance will evaluate the potential risk for GBS to guide ACIP recommendations.

## Abstract

In May 2023, the Food and Drug Administration (FDA) licensed Arexvy and Abrysvo vaccines for prevention of respiratory syncytial virus (RSV) lower respiratory tract disease in adults aged ≥60 years. In prelicensure trials, Guillain-Barré syndrome (GBS) was identified as a potential safety concern. During August 4, 2023–March 30, 2024, at least 10.6 million adults aged ≥60 years received a recommended RSV vaccine. During May 3, 2023–April 14, 2024, CDC reviewed data reported after RSV vaccination to V-safe, an active U.S. surveillance system that invites enrolled participants to complete web-based surveys, and reports to the Vaccine Adverse Event Reporting System (VAERS), a passive, voluntary surveillance system that accepts adverse event reports from the public, providers, and manufacturers. Findings from V-safe and VAERS were generally consistent with those from trials. Reporting rates of GBS after RSV vaccination in VAERS (4.4 and 1.8 reports per million doses of Abrysvo and Arexvy vaccine administered, respectively) were higher than estimated expected background rates in a vaccinated population. CDC and FDA are conducting population-based surveillance to assess risks for GBS and other adverse events. Findings from these studies will help guide development of Advisory Committee on Immunization Practices recommendations.

## Introduction

Respiratory syncytial virus (RSV) infection can cause lower respiratory tract disease, hospitalization, and death in older adults and is responsible for substantial morbidity and mortality among this age group (*1*). The Food and Drug Administration (FDA) licensed Arexvy (GlaxoSmithKline Biologicals [GSK]) and Abrysvo (Pfizer Inc.) vaccines on May 3 and May 31, 2023, respectively, for prevention of lower respiratory tract disease caused by RSV in adults aged ≥60 years (*2**,**3*). On June 21, 2023, the Advisory Committee on Immunization Practices (ACIP) recommended that adults aged ≥60 years may receive a single dose of RSV vaccine, using shared clinical decision-making (*4*). Guillain-Barré syndrome (GBS) was identified as a potential vaccine safety concern in clinical trials of both RSV vaccines (*4*). To characterize early post-marketing vaccine safety findings in adults aged ≥60 years after RSV vaccination, CDC reviewed health surveys and adverse events reported to V-safe, an active U.S. surveillance system that sends web surveys to enrolled participants during the 6 weeks after vaccination, and the Vaccine Adverse Event Reporting System (VAERS), a passive, voluntary surveillance system that monitors adverse events after vaccination, during May 3, 2023–April 14, 2024* (*5*). During August 4, 2023–March 30, 2024, approximately 7.2 million adults aged ≥60 years received GSK RSV vaccine, and 3.4 million received Pfizer RSV vaccine.^†^ Among the 16,220 V-safe participants aged ≥60 years who reported receiving an RSV vaccine and completed one or more daily surveys, 39.0% reported at least one symptom after vaccination; 0.4% of participants reported receiving medical care. VAERS received 3,200 reports of adverse events after RSV vaccination among persons aged ≥60 years (including 28 verified reports of GBS); 91.2% of reports were classified as nonserious. Estimated VAERS GBS reporting rates after RSV vaccination were 4.4 and 1.8 reports per million administered doses of Pfizer and GSK vaccines, respectively. CDC and the partnership between FDA and the Centers for Medicare & Medicaid Services are conducting population-based surveillance assessments of RSV vaccine safety.

## Methods

V-safe (https://vsafe.cdc.gov) is a voluntary, active U.S. surveillance system that sends web surveys to enrolled participants on days 0–7 after vaccination, based on the reported vaccination date.^§^ V-safe surveys for adults aged ≥60 years who received an RSV vaccine were available starting October 20, 2023. Daily surveys include questions about local injection site and systemic reactions and health impacts experienced.^¶^ Participants reporting medical care for symptoms are also prompted to complete a VAERS report.

VAERS (https://vaers.hhs.gov) accepts reports of adverse events from health care providers, vaccine manufacturers, and members of the public (*5*). Reports to VAERS generally cannot be used to determine causal associations between adverse events and vaccination. Medical Dictionary for Regulatory Activities Preferred Terms (MedDRA PTs) are assigned by VAERS staff members to signs, symptoms, and diagnostic findings in VAERS reports.** Reports of serious events (including death) to VAERS during May 3, 2023–April 14, 2024, and relevant available medical records were reviewed by CDC experts to form a clinical impression of each reported outcome^††^ (*6*). Using selected MedDRA PTs, a search was performed to identify outcomes of interest, including GBS and immune thrombocytopenia (ITP), multiple cases of which were identified in clinical reviews of serious reports.^§§^

Symptoms and health impacts reported during the week after RSV vaccination were described for V-safe participants aged ≥60 years who were vaccinated during May 3, 2023–April 14, 2024, and completed one or more daily surveys. Primary VAERS adverse event reports after RSV vaccination for persons aged ≥60 years were described by serious and nonserious classification and MedDRA PTs.^¶¶^ All analyses were conducted using SAS software (version 9.4; SAS Institute). Reporting rates for GBS reports that met the Brighton Collaboration case definition (*6*) were estimated using available doses administered as the denominator. This activity was reviewed by CDC, deemed not research, and was conducted consistent with applicable federal law and CDC policy.***

### Review of V-safe Data

During May 3, 2023–April 14, 2024, a total of 16,220 V-safe participants aged ≥60 years reported receiving an RSV vaccine and completed at least one daily survey (Table 1). The median age of these participants was 70 years (range = 60–94 years), 9,684 (59.7%) were women, 6,402 (39.5%) received GSK vaccine, 3,882 (23.9%) received Pfizer vaccine, and 5,936 (36.6%) did not know the manufacturer of the vaccine they received. Approximately one third (5,043; 31.1%) of participants reported receiving one or more other vaccines during the same visit; those most commonly reported were COVID-19 (3,370; 20.8%) and influenza (2,630; 16.2%) vaccines. During the week after vaccination, 6,328 (39.0%) participants reported symptoms they considered possibly related to RSV vaccination. Injection site symptoms were reported by 2,808 (43.9%) participants who received GSK vaccine and 787 (20.3%) who received Pfizer vaccine. Most injection site symptoms were mild (3,351; 20.7%) or moderate (1,889; 11.6%) (Table 2). Systemic symptoms were reported by 2,344 (36.6%) who received GSK and 839 (21.6%) who received Pfizer. Most systemic symptoms were mild (1,997; 12.3%) or moderate (2,184; 13.5%). The most frequently reported symptoms after RSV vaccination were pain at or near the injection site (5,026; 31.0%), fatigue or tiredness (3,327; 20.5%), and muscle or body aches (2,843; 17.5%). Among those who reported other symptoms, those most commonly reported were sore throat (54; 0.3%), dizziness (38; 0.2%), and runny nose (38; 0.2%).

**TABLE 1:** Symptoms and health impacts reported to V-safe for persons aged ≥60 years who received a respiratory syncytial virus vaccine, by manufacturer — United States, May 3, 2023–April 14, 2024

Event	% Reporting symptoms or health impact after vaccination* (no.)			
	GSK	Pfizer	Do not know/Cannot recall	Total
**No. of participants**	6,402	3,882	5,936	**16,220**
**Symptoms reported as related to vaccination**	48.6 (3,113)	27.3 (1,058)	36.3 (2,157)	**39.0 (6,328)**
**Injection site symptoms**	43.9 (2,808)	20.3 (787)	31.1 (1,846)	**33.5 (5,441)**
Pain	41.3 (2,641)	17.7 (688)	28.6 (1,697)	**31.0 (5,026)**
Swelling	11.5 (737)	5.6 (217)	8.4 (497)	**8.9 (1,451)**
Redness	10.5 (671)	5.0 (195)	8.1 (478)	**8.3 (1,344)**
Itching	6.4 (412)	4.2 (162)	5.6 (330)	**5.6 (904)**
Underarm swelling or tenderness	2.6 (165)	1.8 (69)	1.4 (84)	**2.0 (318)**
Rash	1.6 (101)	1.0 (38)	1.4 (86)	**1.4 (225)**
**Systemic symptoms**	36.6 (2,344)	21.6 (839)	27.9 (1,656)	**29.8 (4,839)**
Fatigue or tiredness	25.6 (1,640)	13.3 (515)	19.7 (1,172)	**20.5 (3,327)**
Muscle or body aches	22.0 (1,407)	12.5 (484)	16.0 (952)	**17.5 (2,843)**
Headache	19.2 (1,227)	10.6 (413)	13.8 (820)	**15.2 (2,460)**
Fever^†^	13.1 (836)	7.5 (293)	10.7 (636)	**10.9 (1,765)**
Chills	12.1 (772)	5.8 (226)	8.3 (493)	**9.2 (1,491)**
Joint pain	11.8 (756)	6.6 (255)	8.0 (477)	**9.2 (1,488)**
Nausea	5.0 (317)	3.2 (123)	4.2 (249)	**4.2 (689)**
Diarrhea	3.1 (201)	2.1 (80)	2.9 (172)	**2.8 (453)**
Rash	0.5 (32)	0.5 (20)	0.5 (30)	**0.5 (82)**
Vomiting	0.4 (25)	0.3 (13)	0.6 (36)	**0.5 (74)**
Other^§^	3.9 (248)	2.5 (98)	2.8 (168)	**3.2 (514)**
**Health impact**	10.2 (654)	6.2 (239)	8.8 (524)	**8.7 (1,417)**
Unable to complete normal daily activities	9.1 (580)	5.3 (205)	8.1 (479)	**7.8 (1,264)**
Unable to work or attend school	1.9 (119)	1.3 (51)	1.7 (99)	**1.7 (269)**
Care from health care professional^¶^	0.5 (29)	0.5 (19)	0.3 (20)	**0.4 (68)**
Office visit or urgent care	0.2 (14)	0.3 (12)	0.2 (11)	**0.2 (37)**
Telehealth	0.2 (10)	0.1 (2)	0.1 (3)	**0.1 (15)**
Emergency department	0.03 (2)	0.1 (3)	0.1 (7)	**0.1 (12)**
Hospitalization	0.02 (1)	0.03 (1)	0.03 (2)	**0.02 (4)**
Other	0.1 (8)	0.1 (2)	0 (—)	**0.1 (10)**

* Percentage of participants who reported a symptom or health impact at least once during days 0–7 postvaccination.

^†^ Fever is a self-reported symptom and might not reflect the clinical definition of fever.

^§^ Among those who reported “Other” symptoms, 409 selected additional symptoms from a dropdown menu; most commonly selected were sore throat (54), dizziness (38), runny nose (38), cough (27), dizziness upon standing (17), and congestion (16).

^¶^ Participants can select from more than one type of care received from a health professional, including doctor appointment or urgent care clinic visit, telehealth, virtual health, or email health consultation, emergency department or emergency department visit, hospitalization, and other.

**TABLE 2:** Self-reported symptom severity reported to V-safe for persons aged ≥60 years who received a respiratory syncytial virus vaccine — United States, May 3, 2023–April 14, 2024

Symptom severity*	% Reporting symptoms or health impact after vaccination^†^ (no.)			
	GSK	Pfizer	Do not know/Cannot recall	Total
**No. of participants**	6,402	3,882	5,936	**16,220**
**Any injection site symptoms**	43.9 (2,808)	20.3 (787)	31.1 (1,846)	**33.5 (5,441)**
Mild	27.2 (1,739)	12.4 (482)	19.0 (1,1130)	**20.7 (3,351)**
Moderate	15.3 (982)	7.0 (271)	10.7 (636)	**11.6 (1,889)**
Severe	1.4 (87)	0.9 (34)	1.3 (80)	**1.2 (201)**
**Any systemic symptoms** ^§^	36.6 (2,344)	21.6 (839)	27.9 (1,656)	**29.8 (4,839)**
Mild	15.6 (1,001)	9.0 (350)	10.9 (646)	**12.3 (1,997)**
Moderate	16.4 (1,048)	9.6 (372)	12.9 (764)	**13.5 (2,184)**
Severe	3.8 (242)	2.2 (86)	3.5 (209)	**3.3 (537)**
**Fever or feverish** ^¶^	13.1 (836)	7.5 (293)	10.7 (636)	**10.9 (1,765)**
No recorded temperature	8.2 (525)	4.5 (174)	7.1 (423)	**7.1 (1,122)**
Normal or subfebrile	3.3 (209)	2.0 (78)	2.2 (129)	**2.6 (416)**
Mild	1.5 (96)	1.0 (38)	1.3 (80)	**1.3 (214)**
Moderate	0.1 (6)	0.1 (3)	0.1 (4)	**0.1 (13)**
Severe	0 (—)	0 (—)	0 (—)	**0 (—)**

* Symptom severity was self-reported. The following definitions describe the severity of symptoms: mild = symptoms noticeable, but not problematic; moderate = symptoms limit normal daily activities; or severe = symptoms make daily activities difficult or impossible. Some symptoms have specific severity definitions.

^†^ Percentage of participants who reported a symptom or health impact at least once during days 0–7 postvaccination.

^§^ The symptom severity total differs from the total systemic symptoms reported because severity is not collected for other symptoms.

^¶^ “Fever or feverish” is a self-reported symptom and might not correspond to a clinical definition of fever. The number of registrants (1,765) who reported having a fever or feeling feverish differs from the total who entered information about temperature (643). Severity of fever was defined as follows: normal or subfebrile = 96.0°–100.3°F (35.6°–37.9°C); mild = 100.4°–102.2°F (38.0°–39.0°C); moderate = 102.3°–103.9°F (39.1°–39.9°C); and severe = 104.0°–107.0°F (40.0°–41.7°C).

During the week after vaccination, 1,264 (7.8%) participants reported that they were unable to complete their normal daily activities because of the reported symptoms; 68 (0.4%) reported receiving medical care for the reported symptoms. Among those who reported receiving medical care, five completed a report to VAERS; events reported were chalazion, lower than normal blood pressure, exacerbation of chronic obstructive pulmonary disease, injection site pain, and suspected lichen planus.

### Review of VAERS Data

During May 3, 2023–April 14, 2024, VAERS received and processed 3,200 reports of adverse events among persons aged ≥60 years who reported receiving an RSV vaccine (Table 3),^†††^ including 2,193 (68.5%) for GSK vaccine, 919 (28.7%) for Pfizer, and 88 (2.8%) for which the vaccine manufacturer was unknown. The median age of persons for whom a VAERS report was received was 72 years (range = 60–112 years), and 2,237 (69.9%) reports were for women. At least one other vaccine was received at the same visit for approximately one third (1,050; 32.8%) of reports, with influenza vaccine administered most commonly (625; 19.5%). Among the 3,200 VAERS reports, 346 (10.8%) specified a vaccination error (e.g., product administered at an inappropriate site, extra dose administered, or incorrect route of product administration); 64 (2.0%) reports also indicated that an adverse health event had occurred. Overall, 2,919 (91.2%) reports were classified as nonserious, including 2,026 (92.5%) after receipt of GSK vaccine and 821 (89.1%) after receipt of Pfizer vaccine. Commonly reported events included pain in an extremity (384; 13.2%), headache (376; 12.9%), pain (373; 12.8%), injection site pain (370; 12.7%), and fatigue (355; 12.2%).

**TABLE 3:** Events reported to the Vaccine Adverse Event Reporting System for persons aged ≥60 years after receipt of a respiratory syncytial virus vaccine — United States, May 3, 2023–April 14, 2024

Event	Vaccine, no. reporting (%)			
	GSK	Pfizer	Do not know/Cannot recall	Total
**Total participants**	**2,193**	**919**	**88**	**3,200**
Events among nonserious reports*^,†^	2,026 (92.5)	821 (89.1)	72 (81.8)	**2,919 (91.2)**
Arthralgia	183 (9.0)	85 (10.4)	7 (9.7)	**240 (8.2)**
Erythema	186 (9.2)	57 (6.9)	4 (5.6)	**384 (13.2)**
Fatigue	235 (11.6)	102 (12.4)	18 (25.0)	**355 (12.2)**
Fever	215 (10.6)	83 (10.1)	9 (12.5)	**247 (8.5)**
Headache	261 (12.9)	105 (12.8)	10 (13.9)	**376 (12.9)**
Injection site erythema	261 (12.9)	66 (8.0)	2 (2.8)	**275 (9.4)**
Injection site pain	291 (14.4)	72 (8.8)	7 (9.7)	**370 (12.7)**
Injection site swelling	187 (9.2)	51 (6.2)	2 (2.8)	**376 (12.9)**
Pain	276 (13.6)	85 (10.4)	12 (16.7)	**373 (12.8)**
Pain in extremity	282 (13.9)	94 (11.4)	8 (11.1)	**384 (13.2)**
**Events among serious reports^§,¶^**	167 (7.6)	98 (10.7)	16 (18.2)	**281 (8.8)**
Allergic reaction**	3	4	0	**7**
Anaphylaxis	1	1	0	**2**
Arrhythmia, other	4	1	1	**6**
Atrial fibrillation^††^	8	3	3	**14**
Congestive heart failure	2	2	0	**4**
Dyspnea or cough	3	2	0	**5**
Encephalitis or aseptic meningitis	5	5	1	**11**
Guillain-Barré syndrome^§§^	18	19	0	**37**
Injection site pain or reaction^¶¶^	4	0	0	**4**
Immune thrombocytopenia***	5	6	0	**11**
Myocardial infarction	3	1	0	**4**
Pneumonia	5	3	1	**9**
Rash	1	2	1	**4**
RSV infection	3	2	0	**5**
Sepsis, bacteremia, or both	6	5	0	**11**
Shoulder pain	7	1	3	**11**
Stroke or transient ischemic attack	13	10	1	**24**
Syncope	6	1	0	**7**
Thromboembolic event, other^†††^	7	4	2	**13**
Transverse myelitis	2	1	0	**3**
Unevaluable	2	2	0	**4**
Death^§§§^	22	11	2	**35**

**Abbreviations:** GBS = Guillain Barré syndrome; MedDRA PT = Medical Dictionary for Regulatory Activities Preferred Term; RSV = respiratory syncytial virus; VAERS = Vaccine Adverse Event Reporting System.

* Each event is a sign or symptom in a VAERS report, coded by a MedDRA PT. MedDRA PTs are assigned by VAERS staff members after review of available data. Each VAERS report might be assigned more than one MedDRA PT, which can include normal diagnostic findings. A MedDRA PT does not necessarily indicate a medically confirmed diagnosis.

^†^ Includes the top 10 most frequently coded MedDRA PTs among nonserious reports.

^§^ VAERS reports are classified as serious if any of the following are reported: hospitalization (216), prolongation of hospitalization (three), life-threatening illness (81), permanent disability (66), congenital anomaly or birth defect (zero), or death (34).

^¶^ Serious reports to VAERS were reviewed by CDC physicians and experts to form preliminary clinical impressions. Includes 20 most common events from preliminary clinical impressions; a report might include more than one event. Because of the small number of serious reports, percentages are not provided for serious report events. Other clinical impressions included acute appendicitis, acute hepatitis, acute on chronic renal failure, acute on chronic respiratory failure, acute renal failure, altered mental status (three), anti-neutrophil cytoplasmic antibody vasculitis, angina, autoimmune hemolytic anemia, body temperature fluctuation, cellulitis in leg, chest pain (three), choked, chronic pulmonary fibrosis, chronic obstructive pulmonary disease (two), COVID-19 infection, duodenal ulcer, epidural abscess, episodic memory loss, fall, fever (two), generalized weakness (three), headache (two), hypertension (two), hypoglycemia, laryngospasm, lower extremity ischemia, myalgia (two), myocarditis, nausea and vomiting, osteoarthritis (three), pancreatitis, pancytopenia, polymyalgia rheumatica, posterior reversible encephalopathy syndrome, reactive arthritis, receptive aphasia, respiratory distress, rhabdomyolysis, subdural hematoma after fall, seizure, spinal stenosis post laminectomy, stress cardiomyopathy, systemic inflammatory response syndrome, third cranial nerve palsy, tinnitus and hearing loss (three), unevaluable (four), urinary tract infection, vaccination related anxiety, viral illness with delirium, and visual impairment.

** Includes two reports of angioedema.

^††^ Includes eight reports of new-onset atrial fibrillation.

^§§^ This review of reports to VAERS includes 21 of the 23 verified GBS reports after RSV vaccination (including one report for a person who died) presented at the February 29, 2024, Advisory Committee on Immunization Practices meeting. Excluded from this report were one report for a person aged 50 years and one report that did not include RSV vaccine in the primary report. In addition, seven reports did not meet the case definition or were unverified because of a lack of records, and two reports remain under review. Three additional unverified reports were identified using the selected MedDRA PTs search and are not included in the table.

^¶¶^ Includes one report of pyoderma.

*** Two additional nonserious reports were identified using the selected MedDRA PTs search, which are not included in the table.

^†††^ Includes reports of pulmonary embolism (10), deep vein thrombosis (two), and retinal artery occlusion (one).

^§§§^ For reports of death, the following reported causes of death was available for 18 reports: acute respiratory distress syndrome, bronchopneumonia, cardiac event, cardiopulmonary arrest, ehrlichiosis, GBS (two), hepatic encephalopathy, hypoxic respiratory failure, multifocal leukoencephalopathy, respiratory failure, rhabdomyolysis, RSV infection, sepsis, sepsis secondary to pneumonia, septic shock (*Pseudomonas* bacteremia), varicella-zoster virus meningoencephalitis, and vascular dementia.

Among all VAERS reports, 281 (8.8%) were classified as serious, including 216 (6.8%) for hospitalization, 81 (2.5%) for a life-threatening illness, 66 (2.1%) for a permanent disability, and 34 (1.1%) for death. Clinical impressions of serious reports included stroke or transient ischemic attack (24), GBS (37; 28 met case definition),^§§§^ atrial fibrillation (14), other thromboembolic event (13), encephalitis or aseptic meningitis (11), immune thrombocytopenia (11), sepsis, bacteremia, or both (11), and shoulder pain (11). Among the 28 reports of GBS after vaccination that met case definition, 13 (46.4%) were after GSK vaccine (1.8 reports per 1 million doses administered), and 15 (53.6%) were after Pfizer vaccine (4.4 reports per 1 million doses administered). For the 18 reports of death with sufficient information for review, reported causes of death were acute respiratory distress syndrome, bronchopneumonia, cardiac event, cardiopulmonary arrest, ehrlichiosis, GBS (two), hepatic encephalopathy, hypoxic respiratory failure, multifocal leukoencephalopathy, respiratory failure, rhabdomyolysis, RSV infection, sepsis, sepsis secondary to pneumonia, *Pseudomonas* bacteremia, varicella-zoster virus meningoencephalitis, and vascular dementia.

## Discussion

This review provides early findings from V-safe and VAERS surveillance systems during the first months of GSK and Pfizer RSV vaccine administration among U.S. adults aged ≥60 years. The findings in this report are generally consistent with those from safety data collected in prelicensure clinical trials, including the observance of GBS cases^¶¶¶^ (*2*,*3*).

In V-safe, injection site and systemic reactions were more frequently reported among those who received GSK than among those who received Pfizer vaccine; few participants reported receiving medical care (*2**,**3*). Expected vaccination reactions (e.g., pain in extremity, headache, and fatigue) were among the most frequently reported events among nonserious VAERS reports. Using VAERS data, estimated GBS reporting rates after RSV vaccination among persons aged ≥60 years were 4.4 and 1.8 reports per million doses of Pfizer and GSK vaccine administered, respectively.

VAERS reporting rates of GBS after mRNA COVID-19 vaccination were used to estimate expected background rates of GBS in this study population; no excess risk for GBS was observed after mRNA COVID-19 vaccinations in active Vaccine Safety Datalink surveillance (*7*). VAERS reporting rates for GBS among adults aged ≥65 years were 0.43 and 0.54 per million doses of Pfizer-BioNTech and Moderna COVID-19 vaccines, respectively**** (*8*). Thus, using the reporting rate for mRNA COVID-19 vaccines as an estimate of background rate, reports of GBS after RSV vaccination were more common than expected. Two deaths among vaccine recipients who had been diagnosed with GBS were reported.

### Limitations

The findings in this report are subject to at least four limitations. First, V-safe is a voluntary program, and data might not be representative of the vaccinated population. Second, VAERS is a passive surveillance system and is subject to reporting biases, underreporting (especially of nonserious events), and incomplete data reporting. Third, VAERS generally cannot determine causal associations between adverse events and vaccination (*5*). Finally, because these data do not include a comparison group of unvaccinated persons with a similar likelihood of receiving an RSV vaccine, estimating the magnitude of risk for serious but rare outcomes (e.g., GBS) after vaccination is not possible.

### Implications for Public Health Practice

On February 29, 2024, ACIP announced that, based on a thorough review of currently available data, the estimated benefits of RSV vaccination continued to outweigh potential risks. RSV vaccination continues to be recommended for adults aged ≥60 years using shared clinical decision-making (*9*). CDC and FDA are conducting active safety evaluations to assess risks for GBS and other adverse events of special interest after RSV vaccination. Results of these studies will help guide future CDC RSV vaccine recommendations.

## Footnotes

* This review includes V-safe data collected during October 20, 2023–April 14, 2024, for persons vaccinated during May 3, 2023–April 1, 2024. This review includes VAERS reports collected during May 3, 2023–April 14, 2024, for persons vaccinated during May 3, 2023–April 2, 2024, and reports that are missing a date of vaccination.

† Projected doses administered during August 4, 2023–March 30, 2024, at physician medical offices (data source: IQVIA Custom Medical Claims [Dx]; data current through April 6, 2024) and during August 12, 2023–March 29, 2024, at retail pharmacies (data source: IQVIA Custom Longitudinal Prescription Claims [LRx]); data are current through April 5, 2024). IQVIA data do not include vaccinations administered at other medical settings such as public health clinics, including workplaces and community locations. IQVIA uses a proprietary methodology to project doses administered in all retail pharmacies and all office-based physicians based on a sample of retail pharmacies and a sample of office-based physicians. The projection to office-based physicians uses a list of U.S.-licensed office-based physicians maintained by the American Medical Association.

§ Registered account holders can add dependents to their accounts and complete surveys on their behalf.

¶ Symptom severity is self-reported as mild (symptoms noticeable, but not problematic), moderate (symptoms limit normal daily activities), or severe (symptoms make daily activities difficult or impossible); some symptoms have additional, specific severity definitions. Participants who report “other” systemic reactions can select signs, symptoms, and health conditions from a dropdown menu. The dropdown menu of 814 common signs, symptoms, and health conditions consists of the most common self-reported MedDRA PTs among VAERS reports.

** Each VAERS report might be assigned more than one MedDRA PT. A MedDRA-coded event does not indicate a medically confirmed diagnosis. https://www.meddra.org/how-to-use/basics/hierarchy

†† VAERS reports are classified as serious (http://www.accessdata.fda.gov/scripts/cdrh/cfdocs/cfcfr/CFRSearch.cfm?fr5600.80) if any of the following events are reported: hospitalization, prolongation of hospitalization, life-threatening illness, permanent disability, congenital anomaly or birth defect, or death. Medical records are requested for reports of serious adverse events, including autopsy findings and death certificates for reported deaths.

§§ CDC experts reviewed primary reports of GBS to VAERS. Reports of GBS within 42 days of RSV vaccination that met the Brighton Collaboration case definition for GBS levels 1–3 were included. Clinical reviews of serious reports identified multiple cases of ITP. To identify other potential ITP cases, a group of MedDRA PTs for thrombocytopenia were used; two additional reports of ITP were detected with this search.

¶¶ A primary VAERS report is the first report of an event after vaccination for a particular patient; subsequent reports pertaining to the same patient and event (from the same reporter or other reporters) are termed secondary reports. Excluded from analysis were reports with missing age (1,064) or age <60 years (388), including reports indicating pregnancy at time of vaccination (401).

*** 45 C.F.R. part 46.102(l)(2), 21 C.F.R. part 56; 42 U.S.C. Sect. 241(d); 5 U.S.C. Sect. 552a; 44 U.S.C. Sect. 3501 et seq.

††† Processed VAERS reports are those that have been coded using MedDRA, deduplicated, and undergone standard quality assurance and quality control review.

§§§ This review of reports to VAERS includes 21 of the 23 verified GBS reports after RSV vaccination presented at the February 29, 2024, ACIP meeting. Excluded from this review were one report for a person aged 50 years and one report that did not include RSV vaccine in the primary report.

¶¶¶ All but one verified VAERS report of GBS indicated that symptoms occurred within 21 days of RSV vaccination. The other report indicated onset of GBS symptoms 22 days after RSV vaccination. In GSK RSV vaccine clinical trials in older adults (18,304 vaccine recipients aged ≥60 years), one case of GBS was reported within 42 days after receipt of the GSK vaccine. In Pfizer RSV vaccine clinical trials in older adults (20,255 vaccine recipients aged ≥60 years), two cases of GBS were reported within 42 days after vaccination.

**** VAERS reports of GBS within 21 days of Pfizer-BioNTech and Moderna COVID-19 vaccination that met the Brighton Collaboration case definition for GBS levels 1–3 were included in reporting rate estimations.
