# *Erratum:* Vol. 73, No. 21

**DOI:** 10.15585/mmwr.mm7327a4

**Published:** 2024-07-11

The report “Early Safety Findings Among Persons Aged ≥60 Years Who Received a Respiratory Syncytial Virus Vaccine — United States, May 3, 2023–April 14, 2024” contained several errors.

On page 489, the sixth sentence in the Abstract should have read, “Reporting rates of GBS after RSV vaccination in VAERS (**4.4** and **1.8** reports per million doses of Abrysvo and Arexvy vaccine administered, respectively) were higher than estimated expected background rates in a vaccinated population.”

On page 489, the fourth complete sentence in the second column should have read, “Estimated VAERS GBS reporting rates after RSV vaccination were **4.4** and **1.8** reports per million administered doses of Pfizer and GSK vaccines, respectively.”

On page 490, the final sentence beginning in the second column should have read, “During May 3, 2023–April 14, 2024, VAERS received and processed 3,200 reports of adverse events among persons aged ≥60 years who reported receiving an RSV vaccine (Table 3),^†††^ including **2,193 **(**68.5**%) for GSK vaccine, **919 **(**28.7**%) for Pfizer, and 88 (2.8%) for which the vaccine manufacturer was unknown.”

On page 492, the first complete sentence should have read, “Among the 28 reports of GBS after vaccination that met case definition, **13** (**46.4**%) were after GSK vaccine (**1.8** reports per 1 million doses administered), and **15** (**53.6**%) were after Pfizer vaccine (**4.4** reports per 1 million doses administered).”

On page 492, the third sentence in the second column should have read, “Using VAERS data, estimated GBS reporting rates after RSV vaccination among persons aged ≥60 years were **4.4** and **1.8** reports per million doses of Pfizer and GSK vaccine administered, respectively.”

On page 493, in Table 3, the total participants for GSK should have read **2,193**, and the total participants for Pfizer should have read **919**. Under “Events among serious reports,” the GSK number and percentage should have read **167 **(**7.6**), and the Pfizer number and percentage should have read **98 **(**10.7**). Under “Guillain-Barré syndrome,” the GSK number should have read **18**, and the Pfizer number should have read **19**.

On page 494, in the Summary, the sentence under “What is added by this report?” should have read, “Findings are consistent with those from trials; reports of GBS (**4.4** and **1.8** reports per million doses of Abrysvo and Arexvy vaccine administered, respectively) were more common than expected background rates.

